# Statin Use and Amyotrophic Lateral Sclerosis Survival: A Population‐Based Cohort Study

**DOI:** 10.1111/ene.70095

**Published:** 2025-03-04

**Authors:** Anders Myhre Vaage, Trygve Holmøy, Jesper Dahl, Hein Stigum, Haakon E. Meyer, Ola Nakken

**Affiliations:** ^1^ Department of Neurology Akershus University Hospital Lørenskog Norway; ^2^ Institute of Clinical Medicine University of Oslo Oslo Norway; ^3^ Department of Infection Control and Vaccines Norwegian Institute of Public Health Oslo Norway; ^4^ Department of Community Medicine and Global Health University of Oslo Oslo Norway; ^5^ Department of Physical Health and Ageing Norwegian Institute of Public Health Oslo Norway

**Keywords:** amyotrophic lateral sclerosis, cohort studies, hydroxymethylglutaryl‐CoA reductase inhibitors, statins, survival

## Abstract

**Background:**

Dyslipidemia is common in amyotrophic lateral sclerosis (ALS). Statin use has been associated with both favorable and poor prognoses. We assessed whether statin use affects ALS survival.

**Methods:**

We linked four Norwegian health surveys (1972–2003) with mandatory national registries to obtain information on premorbid health, ALS diagnosis, and death. Using the Norwegian Prescribed Drug Registry, we identified participants who had dispensed statins pre‐ and post‐diagnosis. We first compared pre‐diagnosis statin discontinuation rates between ALS patients and matched controls. Flexible parametric models were then fitted to estimate the relationship between statin use and survival time in ALS, using restricted mean survival time and hazard ratio (HR) as effect measures.

**Results:**

A total of 524 patients (43% female) with ALS were included. Mean time from ALS diagnosis to death or end of study was 2.0 (SD 2.1) years. A substantial proportion of statin users (21%) discontinued statins during the year leading up to diagnosis. This group was characterized by poorer ALS prognosis compared to those adhering to statins and were included as statin users in our analysis. After adjusting for sex, age, birth year, riluzole use and premorbid smoking status, body mass index, and total cholesterol levels, statin use was not associated with ALS survival. The estimated mean survival difference comparing statin users to non‐users was 0.74 (95% CI −5.98 to 7.47) months, corresponding to a HR of 0.97 (95% CI 0.77–1.23).

**Conclusion:**

Statin use was not associated with ALS survival, suggesting that statins should not routinely be discontinued in ALS.

## 1 Introduction

Amyotrophic lateral sclerosis (ALS) is a fatal neurodegenerative disease, with clinical manifestations involving progressive skeletal muscle loss, dysphagia, dysarthria, and respiratory impairment [1]. The median survival of ALS is 2–4 years [1], but 5%–10% are alive 10 years after diagnosis [2]. Higher rates of cardiovascular disease have been reported in ALS [3], and up to 10% of ALS deaths were considered cardiac when assessed clinically and post‐mortem [4]. Dyslipidemia with increased levels of low‐density lipoprotein (LDL) cholesterol and total cholesterol has been observed in both presymptomatic and prevalent ALS patients [5, 6, 7], but it is uncertain whether it can predict ALS survival. Some studies have reported a paradoxically improved ALS survival associated with increased levels of total cholesterol [8, 9], LDL‐cholesterol [8], or triglycerides [10], whereas others did not [11, 12]. A recent study found that muscle cholesterol accumulates in ALS patients and presymptomatic ALS risk gene variant carriers, possibly leading to greater muscle dysfunction [13].

3‐hydroxy‐3‐methylglutaryl‐coenzyme A reductase inhibitors (“statins”) are commonly prescribed to reduce hypercholesterolemia [14], and have demonstrated effects in both primary and secondary prevention of cardiovascular events [15]. Statins are in general considered to be safe. Muscle symptoms are the most commonly reported adverse events, ranging from myalgia and muscle cramps to rare cases of myopathy and rhabdomyolysis [16]. Whether statin use can modify ALS risk remains elusive, as studies have indicated both a beneficial [17] and harmful effect [18], as well as no clear association [19].

The suggested protective effect of dyslipidemia on ALS survival [8], the reduced survival observed in SOD1‐mice treated with statins [20, 21], and reports of increased muscle cramps and functional decline among ALS patients using statins [22] have raised concerns about the safety of statins in ALS. A widely used clinical guideline recommends discontinuing statins upon an ALS diagnosis [23]. Given that the risks of both cardiovascular disease [24] and ALS [25] increase with age, statin use is a common concern in ALS management.

To our knowledge, no randomized controlled trials (RCTs) have been conducted to evaluate the impact of statins on ALS survival. In contrast to RCTs, observational studies of drug use are susceptible to several biases, including confounding by indication [26]. This bias arises when the reason for prescribing a medication (the indication) is associated with the outcome of interest. Consequently, observational studies evaluating the effect of statin use on ALS survival should control for factors that are related to both the propensity of being prescribed statins and ALS survival. In addition to dyslipidemia, for which statins are prescribed [14], cardiovascular risk factors such as body mass index (BMI) [27] and smoking [28] have been linked to ALS survival [29, 30].

Linking cardiovascular risk information from Norwegian health surveys to drug prescription, death, and patient registries, we designed a cohort study where the aim was to assess the relationship between statin use and survival time in ALS.

## 2 Methods

### 2.1 Study Population and Data Sources

Our study population of ALS patients was nested from a cohort of participants initially recruited in former Norwegian health surveys (1972–2003). The Oslo Study (1972–1973) had a 65% attendance rate and included 17,973 men aged 20–49 years in Oslo [31]. The Counties Study (1974–1988) included 94,022 persons aged 35–49 years (90% attendance rate) from three Norwegian Counties (Oppland, Sogn og Fjordane and Finnmark) [32]. The age 40 program (1985–1999) had an average attendance rate of 69%, encompassing 417,097 participants aged 40–45 years nationwide (excluding Oslo) [33]. Cohort of Norway (CONOR) (1994–2003) combined 10 regional cohorts with similar and standardized health data, achieving a 58% attendance rate with 173,236 participants [34]. All the health surveys concentrated on cardiovascular risk factors, and all comprised physical examination, a questionnaire, and a blood test with similar standardized protocols [34]. Measurements included height, weight, blood pressure, and non‐fasting total cholesterol. The participants answered various health‐related questions, including on smoking and alcohol status. Some participants were registered in more than one health survey, and the surveys encompassed 652,523 unique individuals in total.

**FIGURE 1:**
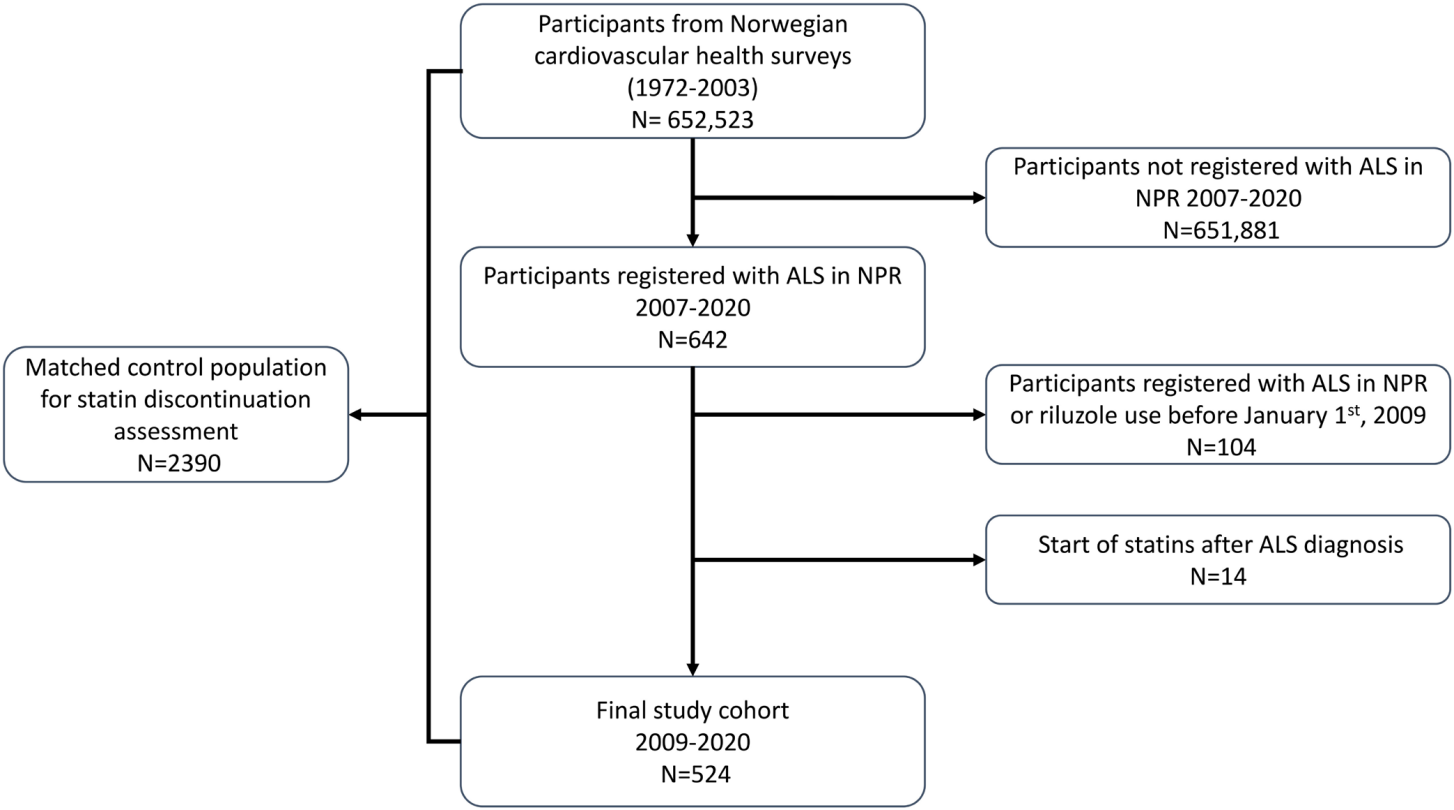
Selection of participants. ALS, amyotrophic lateral sclerosis; NPR, Norwegian patient registry.

Cardiovascular risk information from these health surveys conducted prior to statin use and ALS disease was linked with the Norwegian Patient Registry (NPR) to ascertain ALS disease and time of diagnosis (Figure 1). In NPR, International Classification of Disease (ICD)‐10‐codes are logged for each in‐ and outpatient consultation in hospitals and private practice specialists with public reimbursement. ALS patients in Norway are usually followed and assessed every 3 months in hospital‐based multidisciplinary teams. Data from NPR are available from March 1st, 2007. We defined ALS as having at least two G12.2 registrations (*n* = 642), with the first registration defining the time of diagnosis. This method has demonstrated a high validity in identifying ALS patients in Norway [35]. We aimed to include only incident ALS patients. Therefore, we defined January 1st, 2009, as the start of the capture period in NPR, and participants with one or more G12.2 registrations before this date were excluded (*n* = 103).

The Norwegian Prescribed Drug Registry (NorPD) has information on all dispensed drug prescriptions in Norwegian pharmacies from January 1st, 2004, and onwards. We collected information on the dispensation of statins (Anatomical Therapeutic Chemical [ATC] code C10AA) and riluzole (ATC code N07XX02) from the entire operational period of the registry. We excluded one additional participant who had a first G12.2 registration in NPR after January 1st, 2009, but who had dispensed riluzole prior to this date.

Statins are aimed at long‐term disease prevention. Initiation of a statin after an ALS diagnosis can therefore indicate a false ALS code within NPR. To reduce the risk of possible misclassification of ALS status, we excluded participants with a post‐diagnostic start of statins (*n* = 14). In total, 524 ALS patients were found eligible and included in the study (Figure 1).

To compare pre‐diagnosis statin discontinuation patterns between ALS patients and health survey participants who did not develop ALS, we randomly selected 10 controls from the surveys for each included ALS patient who had ever used statins, matching them by age, sex, and time of diagnosis (Figure 1).

From the Norwegian Cause of Death Registry, we collected information on the time of death. ALS as a cause of death was defined as having ALS (ICD = G12.2) mentioned anywhere on the death certificate. For other causes of death, we only had information on the time of death.

We included data from all registries from their given start date until the end of follow‐up on December 31st, 2020. Linkage between registries was done through personal identification numbers, which are assigned to every Norwegian citizen.

### 2.2 Exposure and Covariates

Statin use was the exposure of interest. The average time from symptom onset to ALS diagnosis is approximately 1 year in Norway [36]. In order to include statin use in this pre‐diagnostic clinical phase, we defined statin exposure as use at or within 1 year prior to the time of diagnosis. This choice was also in agreement with our assessment of statin discontinuation patterns prior to diagnosis. We further restricted the exposure criteria to at least two statin dispensations and a cumulative dispensation of no less than 60 Defined Daily Doses (DDD). These criteria were set to ensure that participants considered statin users had not discontinued the drug during the initiation phase. After fulfilling the exposure criteria, statin users were defined as exposed throughout the observation time, that is, from the time of diagnosis to the end of follow‐up.

As potential confounders, we collected information from the health surveys (1972–2003) on BMI (kg/m^2^), smoking status, and levels of total cholesterol. From NorPD, we collected information on riluzole use. Use of riluzole was defined as at least two dispensed prescriptions.

### 2.3 Statistical Analysis

We hypothesized that many ALS patients had discontinued statins prior to diagnosis, as statin side effects could mimic early ALS disease manifestations, and that those with rapidly progressive disease and less favorable prognosis were overrepresented among this group. To test this, we first assessed the rate of statin discontinuation in the years prior to ALS diagnosis and compared the rates with a control group who did not develop ALS. We selected 10 controls per ALS patient who had ever used statins from the same health surveys, matched on time of diagnosis, age, and sex. In ALS patients, we then compared characteristics between those adhering to statins and those discontinuing statins. Statins were considered discontinued at the time of the last dispensed statin prescription plus the number of DDDs of that prescription.

In our analysis assessing the impact of statin use on ALS survival, the follow‐up time started at the date of ALS diagnosis (first NPR entry) and ended at the date of death or the end of the study (31st of December 2020), whichever came first. The outcome of interest was all‐cause mortality. Descriptive data are presented as mean (standard deviation [SD]) for continuous variables and number (%) for categorical variables. We compared groups according to statin use at baseline by independent *t*‐test (continuous variables) or *χ* ^2^‐test (categorical variables). To calculate restricted mean survival time (RMST), hazard ratio (HR), and confidence intervals, we fitted flexible parametric survival models using the stpm3 package in STATA [37]. The end time point for RMST was set as the total follow‐up time. Time since ALS diagnosis (months) was used as the time variable.

Statin use was defined as a time‐fixed variable, as all statin users had a pre‐diagnostic start of statins. In a basic model, we adjusted for sex, age at diagnosis, birth year, and health screening. Thereafter, we included smoking status, BMI, total cholesterol, and riluzole use as covariates in the fully adjusted model. Riluzole use was defined as a time‐dependent variable. Participants were considered as non‐riluzole users until their first riluzole dispense and regarded as riluzole users for the remainder of the observation time. The data was nearly complete (only missing 0.6% for smoking, 0.2% for BMI and 1.3% for total cholesterol). For graphical purposes, a survival curve was predicted from the fully adjusted model. Both age and sex heterogeneity were tested using the likelihood ratio test by comparing the basic model with separate models that included an interaction term between statin use and age at diagnosis and between statin use and sex. The proportional hazards assumption was tested by comparing the basic model with a model allowing the exposure to interact with time and did not reveal any violations.

Two sensitivity analyses were conducted were we both restricted and expanded the exposure definition. First, we changed the statin exposure definition to ongoing statin use at the time of diagnosis only. ALS patients registered with their date of statin discontinuation before diagnosis were defined as non‐users. Then, to account for a potentially longer time from symptom onset to diagnosis or a persisting effect from pre‐symptomatic statin use on ALS survival, we secondly defined exposure as statin use at or within 2 years prior to the time of diagnosis.

Statistical significance level was set at *p* < 0.05, and all tests were two‐sided. All data management and statistical analyses were conducted in STATA software version 18 (StataCorp, College Station, TX, USA).

### 2.4 Ethics Statement

The study was approved, and a waiver of informed consent was granted by the regional ethics committee (REC South East reference no. 2016/1731). The study complies with the Declaration of Helsinki.

## 3 Results

### 3.1 Statin Discontinuation Before ALS Diagnosis

**FIGURE 2:**
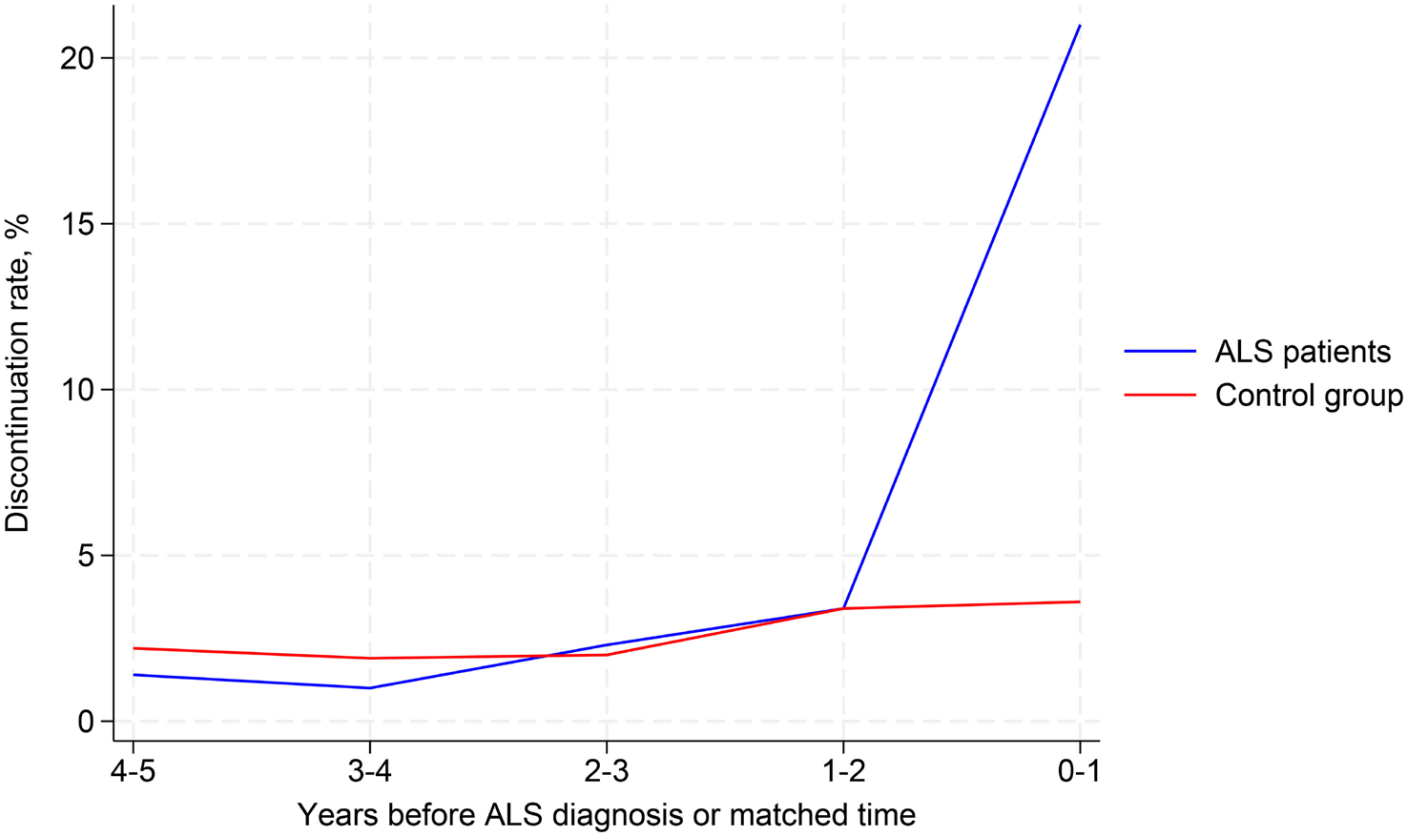
Discontinuation rate of statins among ALS patients compared to the control group matched on sex, age, and time of diagnosis. ALS, amyotrophic lateral sclerosis.

We first assessed the discontinuation of statins in the years prior to ALS diagnosis. During the last year before ALS diagnosis, 40 of 194 ALS patients (21%) discontinued statins. In the second and third years before ALS diagnosis, the annual discontinuation rate was 2%–4%, which was also found in the control group (Figure 2). Those with ongoing statin use at the time of diagnosis were characterized as having better prognosis compared to those who discontinued statins within the year prior to ALS diagnosis. Thus, the ongoing statin users had a longer ALS time under observation (mean 2.0 (SD 2.0) years vs. 1.5 (SD 1.5) years) and comprised more riluzole users (77% vs. 68%). Ultimately, of those who had died during follow‐up, a lower proportion was registered with ALS in death certificates in the group with ongoing statin use compared to those who had discontinued statins (91% vs. 97%).

### 3.2 Statin Use and ALS Survival

Due to the anticipated diagnostic delay in ALS and underpinned by our assessment of pre‐diagnostic statin discontinuation patterns, we defined statin use as use at or within 1 year prior to the time of diagnosis.

A total of 524 persons (43% females) with ALS were followed for a mean of 2.0 (SD 2.1) (maximum 11.8) years. The mean age at diagnosis was 67.0 (SD 8.1) years. Statin users were older and had higher levels of total cholesterol at the health survey compared to non‐users. Characteristics of the study population are presented in Table 1.

**TABLE 1:** Cohort characteristics by statin use.

	Statin use^a^	Non statin use	*p*
Participants	194	330	
Female	77 (39.7%)	150 (45.5%)	0.199
Age at ALS diagnosis	68.8 (7.5)	66.0 (8.2)	< 0.001
Observation years	1.9 (1.9)	2.1 (2.2)	0.147
Birth year	1946 (7.9)	1948 (8.0)	< 0.001
Riluzole use	145 (74.7%)	239 (72.4%)	0.563
Covariates collected from prior health surveys			
Age at health survey	42.8 (SD 7.6)	42.4 (SD 7.2)	0.264
BMI	25.3 (3.8)	25.0 (3.5)	0.153
Total cholesterol in mmol/L	6.3 (1.1)	5.5 (1.0)	< 0.001
Smoking status			
Never	79 (40.9%)	164 (50%)	0.107
Previous	36 (18.7%)	58 (17.7%)	
Current	78 (40.4%)	106 (32.3%)	

*Note:* Mean (SD) for continuous variables and *n* (%) for categorical variables.

Abbreviations: ALS, amyotrophic lateral sclerosis; BMI, body mass index.

^a^ Statin use is defined as the use of statins at or within 1 year prior to the time of diagnosis.

**FIGURE 3:**
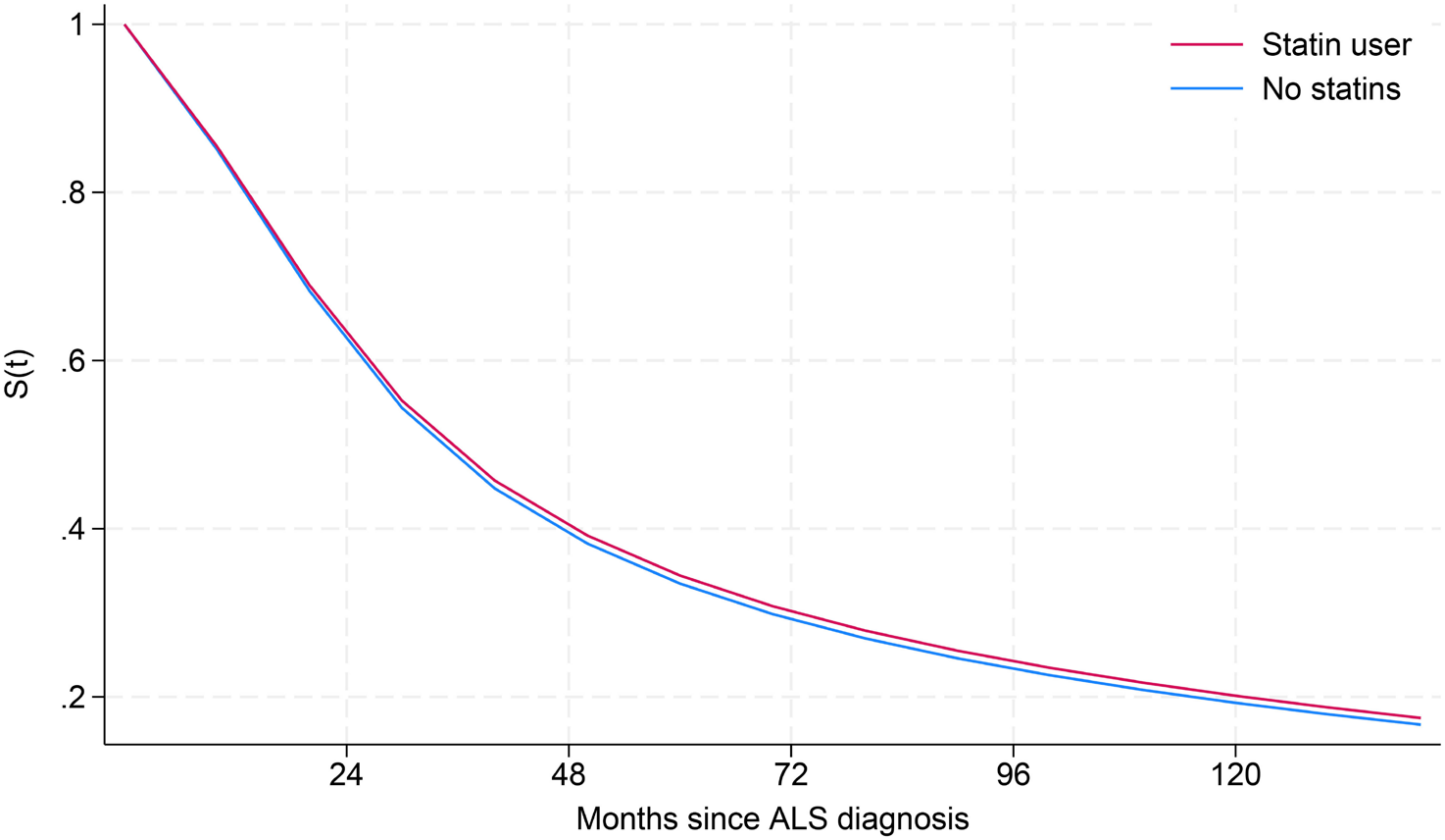
ALS survival curve for statin users versus non‐statin users, adjusted for sex, age at diagnosis, birth year, health survey, body mass index, smoking status, total cholesterol, and riluzole use. ALS, amyotrophic lateral sclerosis; S(t), survival function.

In the fully adjusted model, there was no difference in survival time between statin users and non‐users. The estimated mean survival difference was 0.74 (95% CI −5.98 to 7.47) months (Table 2, Figure 3), corresponding to a hazard ratio (HR) of 0.97 (95% CI 0.77–1.23) for statin users compared to non‐users. There was no evidence of effect modification by sex (*p* = 0.28) or age at diagnosis (*p* = 0.26). In the same model, no association between levels of total cholesterol at the health survey and ALS survival was detected. Thus, HR according to one‐unit increment in total cholesterol was 1.01 (95% CI 0.92–1.12).

**TABLE 2:** Amyotrophic lateral sclerosis survival by statin use.

Medication	Participants	Deaths	Person‐time, months	RMST difference (95% CI), months Model 1	RMST difference (95% CI), months Model 2
Non statin use	322	229	8186	Reference	Reference
Statin use^a^	191	146	4408	−0.99 (−8.64 to 6.66)	0.74 (−5.98 to 7.47)

*Note:* Model 1: Adjusted for sex, age at diagnosis, birth year and health survey. Model 2: Adjusted for sex, age at diagnosis, birth year, health survey, body mass index, smoking status, total cholesterol, and riluzole use.

Abbreviation: RMST, restricted mean survival time.

^a^ Statin use is defined as the use of statins at or within 1 year prior to the time of diagnosis.

In the first sensitivity analysis, statin use was restricted to those with ongoing use at the time of diagnosis. Even though effect estimates were drawn towards a favorable outcome for statin users, no statistically significant associations were found. The mean survival difference between statin users and non‐users was 2.89 (95% CI −3.94 to 9.72) months (Table S1), which corresponded to an HR of 0.90 (95% CI 0.71–1.15) for statin use.

In the second sensitivity analysis, the definition of statin use was expanded to include use at or within 2 years prior to the time of diagnosis. Again, statin use was not associated with ALS survival. The survival difference between statin users and non‐users was −0.03 (95% CI −6.90 to 6.85) months (Table S2), which corresponded to an HR of 1.00 (95% CI 0.79–1.26) for statin use.

## 4 Discussion

In this population‐based cohort study, we found no association between statin use and ALS survival. Notably, a large proportion of statin users discontinued statins in the year prior to ALS diagnosis, possibly due to early disease manifestations mimicking statin side effects. Our data indicated that there were differences in those who discontinued and those who persisted in using statins after diagnosis. Compared to the former, the latter group was characterized by a longer survival time, more riluzole use, and less frequent ALS diagnoses registered in death certificates. To avoid a skewed study population of statin users with more slowly progressive ALS disease, we therefore also included those who used statins a year prior to ALS diagnosis as statin users in our main analysis. In the absence of relevant randomized controlled trials, this cohort study serves an important role in suggesting that statin use does not affect ALS survival.

Our findings are consistent with two previous cohort studies [38, 39]. None of these studies had information on cholesterol values. Both studies [38, 39] calculated survival time and assessed statin use from symptom onset, whereas in the current study survival time was calculated from the date of diagnosis. Time from symptom onset to ALS diagnosis is approximately 1 year [40, 41], also in Norway [36]. Our exposure definition, including statin users in the adjacent year prior to diagnosis, therefore mirrors what was defined as statin exposure in these previous cohort studies. In some individuals, prodromal ALS disease manifestations can occur several years before diagnosis [42]. We therefore tested an expanded statin exposure definition, also including those who discontinued statins up to 2 years before diagnosis. The results were similar to those from our main analysis, strengthening the basis of our conclusions.

One cohort study [22] reported increased functional decline with statin use. This study differed from ours both with regard to ALS ascertainment and statin use definition. Importantly, the study included only a small number of statin users (32), who were generally much older than the controls [22]. Two studies on simvastatin in SOD1‐mice demonstrated increased functional decline and reduced survival [20, 21]; another study on lovastatin in SOD1‐mice indicated prolonged survival [43]. The latter study [43] attributed the differing results to the use of a lower and more appropriate human equivalent dose of statins and initiation before the onset of motor symptoms, contrasting the studies that showed decreased survival [20, 21]. A meta‐analysis [44] and a mendelian randomization study [12] found no clear association between statin use and ALS survival in humans.

A case–control study nested in a large Israeli population found prolonged survival in short‐term ALS statin users, defined as pre‐diagnostic statin use under 3 years, and users of low‐potency statins, but not long‐term or high‐potency use [45]. The study assessed both ALS survival and ALS risk. In order to avoid a false association between statin use and ALS risk due to statin initiation secondary to prodromal ALS, the authors excluded the use of statins 3 years before diagnosis altogether. Although this is a sensible choice when assessing ALS risk, uncertainty regarding persisting or immediate effects makes it less clear in ALS survival.

We found no association between total cholesterol measured several years prior to ALS diagnosis and ALS survival. Although a Norwegian study tracking total cholesterol over 16 years suggests relatively stable levels [46], cholesterol levels may have changed substantially before disease onset. Notably, higher levels of total cholesterol [8], LDL‐cholesterol [8], and triglycerides [10] at or shortly after diagnosis have been associated with longer survival in ALS patients, arguing against adherence to statin therapy. However, discontinuing statins might have unintended consequences, as cardiovascular comorbidities can lead to shorter survival [47]. Further, the association between dyslipidemia and ALS survival remains uncertain. A population‐based case–control study from Germany found that the protective effect of lipids could be mostly attributed to triglycerides, while both LDL‐cholesterol and high‐density lipoprotein (HDL) cholesterol were associated with higher mortality [10]. Triglyceride levels have a smaller impact on, and are less affected by, statin initiation compared to cholesterol levels. A recent combined meta‐analysis, cohort study, and assessment of polygenic profile scores [11], found no association between total cholesterol, LDL‐cholesterol, or triglycerides and ALS survival time. Based on our results, statins should not routinely be discontinued upon ALS diagnosis.

The strengths of this study are the population‐based design with validated methods of case ascertainment and recording of exposure and confounding factors prior to diagnosis, avoiding recall bias. Observational studies in pharmacoepidemiology can be prone to confounding by indication [26]. We addressed this by including the most relevant factors predicting both the propensity of being prescribed statins and ALS survival in our models. Importantly, many of these factors were objectively measured and collected prior to statin initiation. Further, we used a prescription database to ascertain statin use, in contrast to self‐reported drug use, which can be susceptible to recall bias and a higher risk of misclassification.

This study has limitations. The information from the health surveys on total cholesterol, smoking status, and BMI was collected years before ALS diagnosis, and it is unclear how these data agreed with corresponding levels at the time of ALS diagnosis. The total cholesterol levels were measured before statin initiation in almost all cases, affecting both ALS risk [48] and the propensity of being prescribed statins. A later increase in cholesterol levels as a secondary phenomenon can potentially affect ALS survival [8] as well as result in statin initiation closer to diagnosis. Still, we included both long‐term and short‐term statin use, minimizing any confounding from changing cholesterol levels. Further, NorPD only records prescriptions from pharmacies, and not drugs given in hospitals or nursing homes. Some of the more rapidly progressive ALS patients may have been transferred to an institution directly after diagnosis. Although this is rare, it could have biased the sensitivity analysis restricting the statin use definition to those with ongoing use at the time of diagnosis. As statin users were defined also by pre‐diagnostic use in our main analysis, and a sensitivity analysis including statin users 2 years ahead of diagnosis agreed with the main finding, admission to nursing homes due to ALS has probably not affected our overall conclusions. We did not have phenotypic nor genotypic data, preventing stratified analyses on bulbar versus spinal and sporadic versus gene‐driven ALS. The participants in the current study were originally volunteers in health surveys, which may not always be representative of the general population. However, the current health surveys have been shown to be representative of important factors like smoking, alcohol, and educational level [49, 50]. Ultimately, the participants were mostly in their 40s when enrolled in these surveys conducted long before the start of follow‐up. As a result, we could not include younger ALS patients. Still, our study captured patients in the most relevant age groups with regard to both ALS risk and statin use.

In conclusion, statin use was not associated with ALS survival in the current study. This suggests that statin use should not routinely be discontinued upon diagnosis but rather be evaluated based on a patient's overall clinical context.

## Author Contributions

**Anders Myhre Vaage:** writing – original draft, methodology, writing – review and editing, formal analysis. **Trygve Holmøy:** conceptualization, methodology, writing – review and editing. **Jesper Dahl:** methodology, writing – review and editing. **Hein Stigum:** methodology, writing – review and editing. **Haakon E. Meyer:** methodology, writing – review and editing. **Ola Nakken:** conceptualization, methodology, writing – review and editing, supervision.

## Conflicts of Interest

Ola Nakken has received speakers honoraria from Novartis. Trygve Holmøy has received honoraria/consultancy fees from Biogen Idec, Merck, Roche, Bristol Myers Squibb, Santen, Amgen, Novartis, and Sanofi‐Genzyme. Anders M. Vaage, Jesper Dahl, Hein Stigum, and Haakon E. Meyer declare no conflicts of interest.

## Supporting information


Table S1.

Table S2.

## Data Availability

According to Norwegian data privacy regulations, the data used in this study are not sharable. However, researchers can apply to the Norwegian Institute of Public Health for access. Approval from the Norwegian Committee for Medical and Health Research Ethics is required, and any access or use must comply with Norwegian data protection legislation.
