# Supplementary material for: Statin Use and Amyotrophic Lateral Sclerosis Survival: A Population‐Based Cohort Study
Source: Eur J Neurol. 2025 Mar 4;32(3):e70095. doi: 10.1111/ene.70095 (PMC11876845; doi:10.1111/ene.70095)
Supplement: Supplementary file 1 — Table S1. [file ENE-32-e70095-s001.docx]

**Supplementary Table 1, Amyotrophic lateral sclerosis survival by statin use at time of diagnosis**

| Medication | Participants | Deaths | Person-time, months | RMST^a^ difference (95% CI), months  Model 1 | RMST difference (95% CI), months  Model 2 |
| --- | --- | --- | --- | --- | --- |
| No statins | 362 | 260 | 8920 | Reference | Reference |
| Statin use | 151 | 115 | 3674 | 1.57 (-6.55 to 9.69) | 2.89 (-3.94 to 9.72) |

Model 1: Adjusted for sex, age at diagnosis, birth year and health survey

Model 2: Adjusted for sex, age at diagnosis, birth year, health survey, body mass index, smoking status, total cholesterol and riluzole use

^a^RMST=Restricted mean survival time
