# Supplementary material for: Statin Use and Amyotrophic Lateral Sclerosis Survival: A Population‐Based Cohort Study
Source: Eur J Neurol. 2025 Mar 4;32(3):e70095. doi: 10.1111/ene.70095 (PMC11876845; doi:10.1111/ene.70095)
Supplement: Supplementary file 2 — Table S2. [file ENE-32-e70095-s002.docx]

**Supplementary Table 2, Amyotrophic lateral sclerosis survival by statin use at or within two years prior to diagnosis**

| Medication | Participants | Deaths | Person-time, months | RMST^a^ difference (95% CI), months  Model 1 | RMST difference (95% CI), months  Model 2 |
| --- | --- | --- | --- | --- | --- |
| No statins | 316 | 224 | 8108 | Reference | Reference |
| Statin use | 197 | 151 | 4486 | -1.84 (-9.47 to 5.79) | -0.03 (-6.90 to 6.85) |

Model 1: Adjusted for sex, age at diagnosis, birth year and health survey

Model 2: Adjusted for sex, age at diagnosis, birth year, health survey, body mass index, smoking status, total cholesterol and riluzole use

^a^RMST=Restricted mean survival time
